# Supplementary material for: Conspiracy Beliefs Are Associated with Lower Knowledge and Higher Anxiety Levels Regarding COVID-19 among Students at the University of Jordan
Source: Int J Environ Res Public Health. 2020 Jul 8;17(14):4915. doi: 10.3390/ijerph17144915 (PMC7399915; doi:10.3390/ijerph17144915)

Supplementary File 2

Supplementary Figures

Supplementary Figure 1. The distribution of the study participants into different Schools at the University of Jordan. IT: King Abdullah II School of Information Technology.

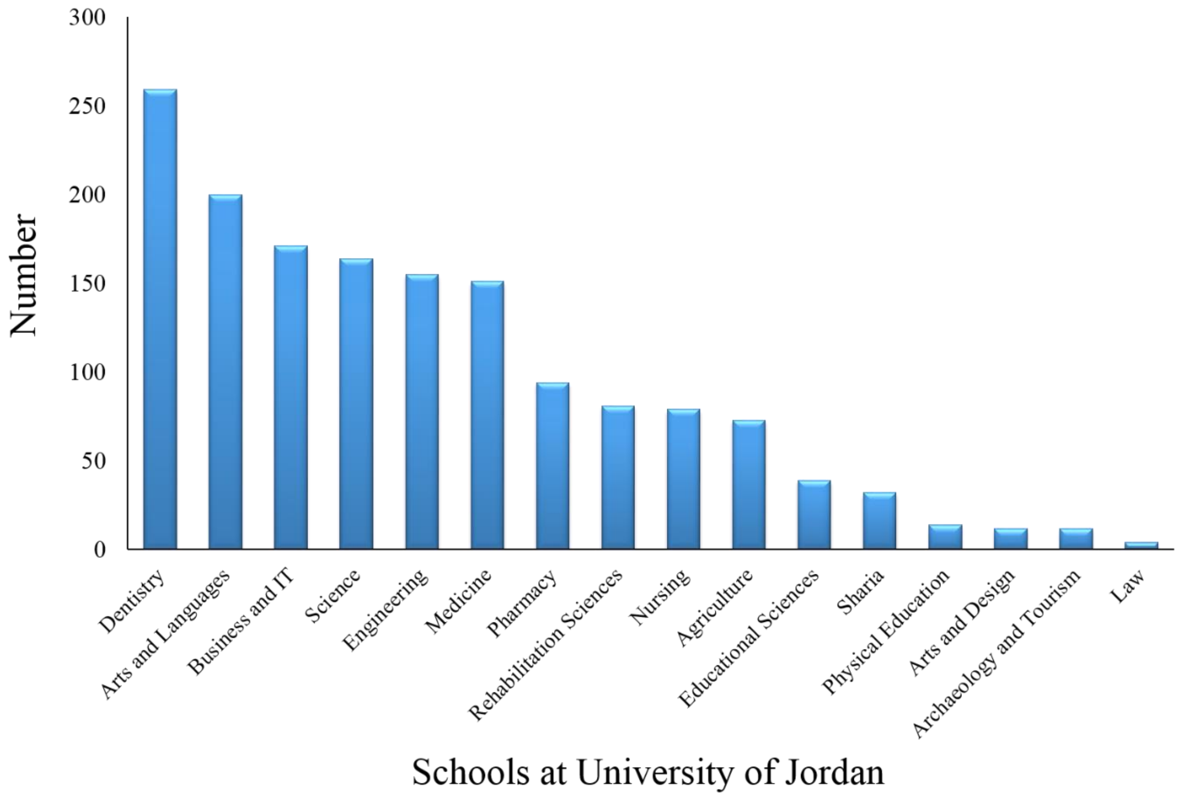

Supplementary Figure 2. The anxiety scores of study participants stratified by monthly income of family. K-W: Kruskal Wallis test; JD: Jordanian dinar; anxiety score calculation was based on 7-item Generalized Anxiety Disorder Scale.

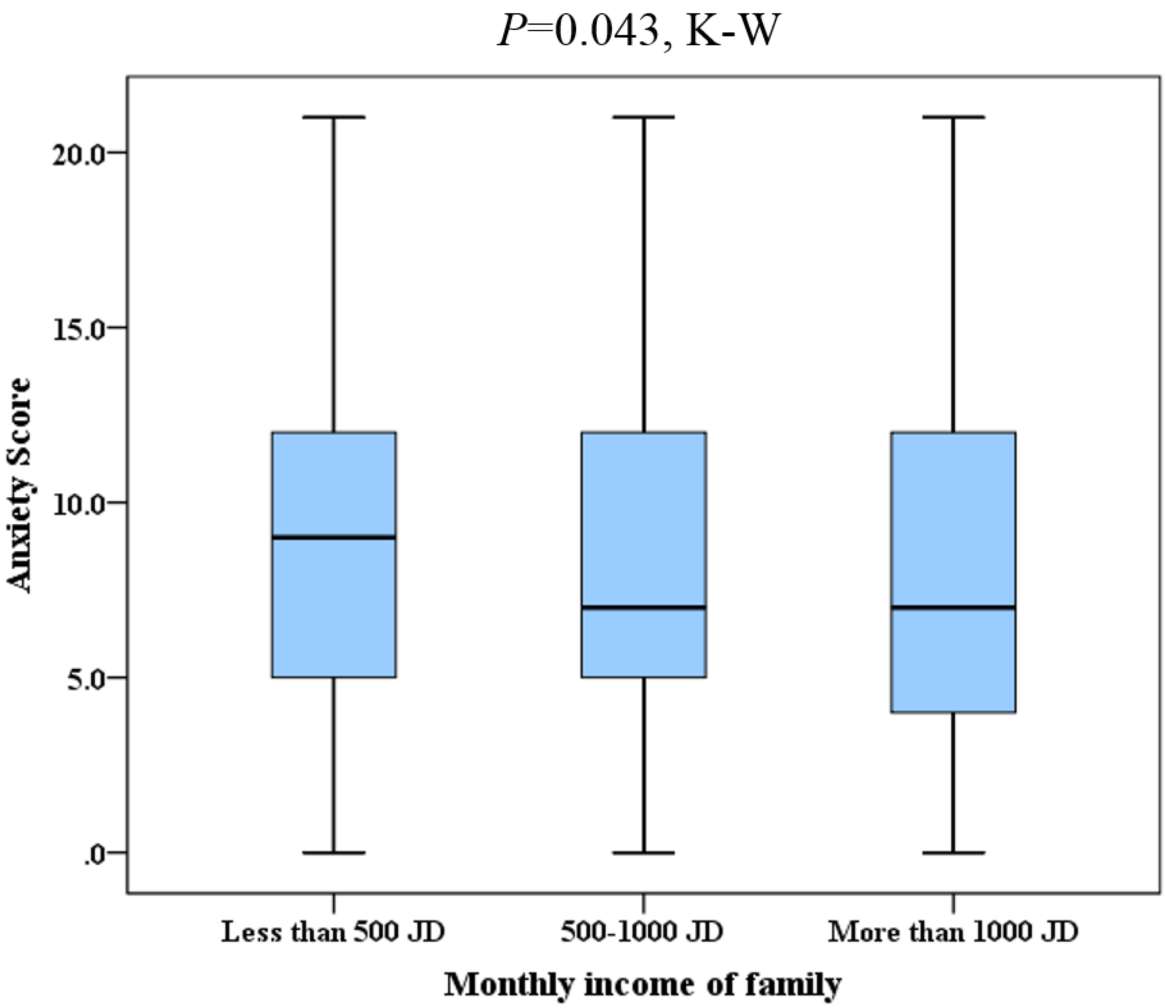

Supplementary Figure 3. The anxiety scores of female study participants divided by nationality. M-W: Mann-Whitney U test; anxiety score calculation was based on 7-item Generalized Anxiety Disorder Scale; participants of non-Jordanian origin included 22 different nationalities, with the most common being Palestine (n=42), Iraq (n=33) and Kuwait (n=28).

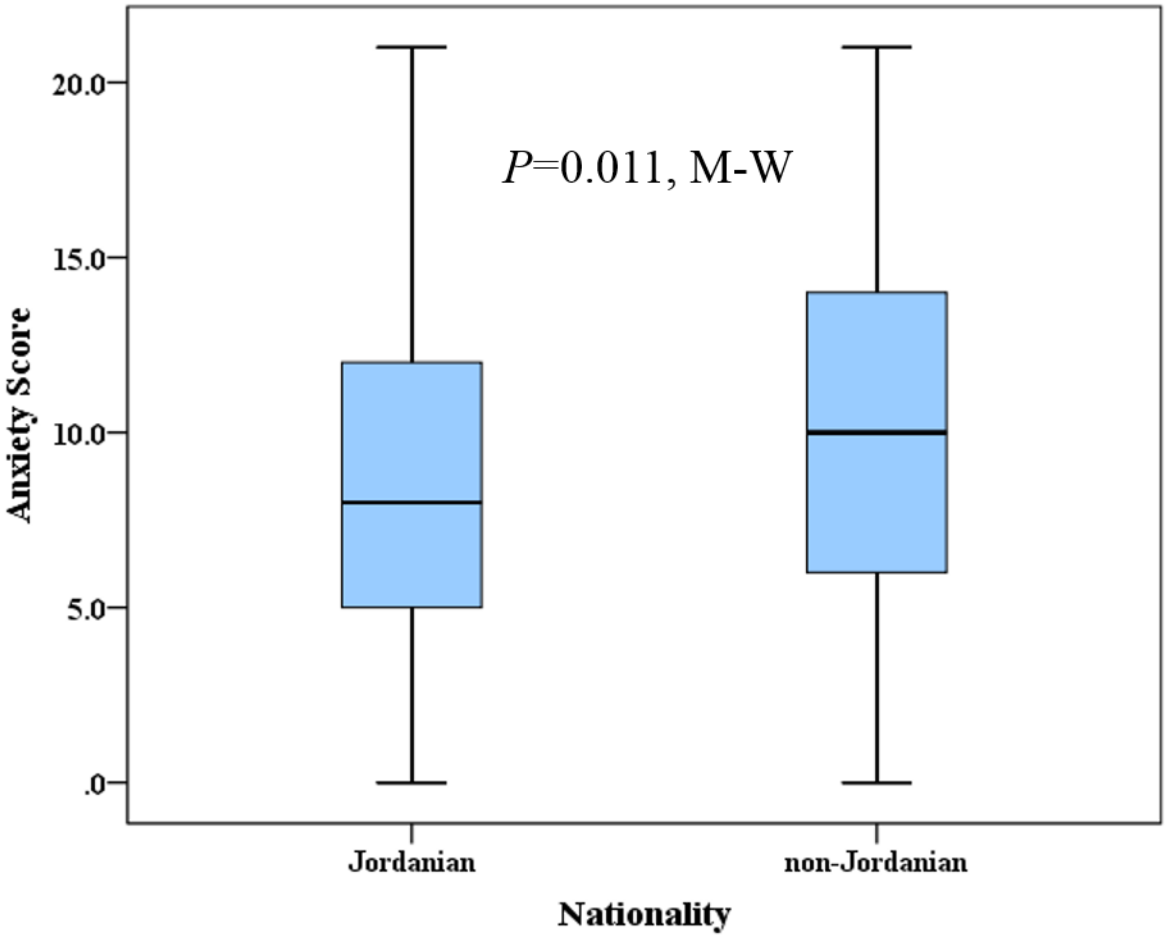

Supplement: Supplementary file 1 [file ijerph-17-04915-s001.zip › Supplementary_File_2.pdf]
