# Supplementary material for: Conspiracy Beliefs Are Associated with Lower Knowledge and Higher Anxiety Levels Regarding COVID-19 among Students at the University of Jordan
Source: Int J Environ Res Public Health. 2020 Jul 8;17(14):4915. doi: 10.3390/ijerph17144915 (PMC7399915; doi:10.3390/ijerph17144915)
Supplement: Supplementary file 1 [file ijerph-17-04915-s001.zip › Supplementary_File_1.pdf]

Supplementary File 1

Consent form and questionnaire translated to English (the original form in Arabic is provided below).

This questionnaire was prepared to measure the extent of knowledge and awareness of students at the University of Jordan about the threats posed by the novel coronavirus.

The information provided to you through this questionnaire will be used for research purposes only.

Participation in this survey is completely voluntary.

Please answer the questionnaire through your current knowledge of the disease, without having to refer to any source or site related to the topic.

Thank you very much for agreeing to participate in this survey.

Age

………………….

Nationality

………………….

Gender

- ☐ Male
- ☐ Female

University Program

- ☐ BSc
- ☐ MSc
- ☐ PhD

School

- ☐ School of Medicine
- ☐ School of Dentistry
- ☐ School of Pharmacy
- ☐ School of Nursing
- ☐ School of Engineering
- ☐ School of Science
- ☐ School of Arts and Design
- ☐ School of Business and King Abdullah II School of Information Technology
- ☐ School of Arts and School of Foreign Languages
- ☐ School of Physical Education
- ☐ School of Agriculture
- ☐ School of Sharia
- ☐ School of Archaeology and Tourism
- ☐ School of Rehabilitation Sciences
- ☐ School of Educational Sciences
- ☐ School of Law

Marital status

- ☐ Single
- ☐ Married
- ☐ Other: ………………….

Monthly income for family

- ☐ Less than 500 Jordanian Dinar
- ☐ 500-1000 Jordanian Dinar
- ☐ More than 1000 Jordanian Dinar

With whom do you spend the quarantine period?

- ☐ Family
- ☐ Other students in dorm
- ☐ By myself
- ☐ Other: ………………….

Is COVID-19 a dangerous disease?

- ☐ Not dangerous
- ☐ Moderately dangerous
- ☐ Very dangerous

You can check multiple boxes

What are the clinical symptoms?

- ☐ Fever
- ☐ Cough
- ☐ Diarrhea and vomiting
- ☐ Shortness of breath
- ☐ Sneezing

The virus is transmitted through:

- Coughing or sneezing
- Touching infected surfaces
- Blood transmission
- Close contact in crowded places

What are the protective methods?

- Wash your hands regularly with soap and water, or clean them with alcohol-based hand rub
- Maintain at least 1 metre distance between you and people coughing or sneezing
- Avoid touching your face
- Cover your mouth and nose when coughing or sneezing
- Stay home if you feel unwell
- Refrain from smoking and other activities that weaken the lungs
- Avoid unnecessary travel
- Stay away from large groups of people

Coronavirus infection can be treated using an antibiotic:

- Yes
- No

There is a vaccine available for COVID-19:

- True
- False

Summer heat can kill the COVID-19 virus:

- True
- False

Where do you get your knowledge about COVID-19 from?

- ☐ Ministry of Health official website
- ☐ Scientific journals
- ☐ Medical Doctors
- ☐ University websites
- ☐ Television programs
- ☐ Social media (Facebook, Instagram, Twitter, WhatsApp)
- ☐ Other: ………………….

If you answered social media, from which platform do you receive your information?

- ☐ Facebook
- ☐ Instagram
- ☐ Twitter
- ☐ WhatsApp
- ☐ Other: ………………….

Do you think the COVID-19 pandemic is part of a global conspiracy theory?

- ☐ Yes
- ☐ No
- ☐ Maybe

Are you adhering to government quarantine rules and staying home?

- ☐ Yes
- ☐ No

Answer the questions regarding how you have been feeling the past 2 weeks of quarantine

Question/*Feeling*	*Not at all*	*Several days*	*More than half the days*	*Nearly everyday*
Are you feeling nervous, anxious, or on edge?	☐	☐	☐	☐
You have trouble relaxing and feeling comfortable	☐	☐	☐	☐
You worry a lot about the number of people infected with the virus	☐	☐	☐	☐
Inability to carry on with daily work	☐	☐	☐	☐
Being so restless and unable to stop thinking	☐	☐	☐	☐
Becoming easily annoyed or irritable	☐	☐	☐	☐
Feeling afraid of this pandemic turning into a global catastrophe.	☐	☐	☐	☐

Thank You

تم اعداد هذا الاستبيان لقياس مدى معرفة طلبة الجامعة الاردنية ووعيهم حول مخاطر فيروس الكورونا المستجد

سيتم استخدام المعلومات المقدمة لك من خلال هذا الاستبيان لأغراض بحثية فقط

المشاركة في هذه الإستبانة طوعية تماماً

شكرا جزيلا للموافقة على المشاركة في هذا الاستطلاع

العمر

الجنسية

الجنس

☒ ذكر

☒ انثى

التخصص

☒ الطب البشري

☒ طب الأسنان

☒ الصيدلة

☒ التمريض

☒ الهندسة

☒ العلوم

☒ الفنون الجميلة

☒ الأعمال و تكنولوجيا المعلومات

☒ الآداب و اللغات

☒ التربية الرياضية

☒ الزراعة

☒ الشريعة

☒ الآثار و السياحة

☒ كلية علوم التأهيل

☒ كلية العلوم التربوية

☒ الحقوق

البرنامج

- البكالوريوس
- الماجستير
- الدكتوراة

الحالة الإجتماعية

- اعزب
- متزوج
- اخرى

الدخل الشهري للأسرة

- أقل من 500 دينار
- بين 500 و 1000 دينار
- أكثر من 1000 دينار

مع من تمضي فترة الحجر الصحي؟

- مع عائلتي
- سكن طلاب
- بمفردي
- اخرى

هل فيروس كورونا المستجد فيروس خطير؟

- ليس خطيراً
- خطير إلى حد ما
- خطيراً جدا

بإمكانك اختيار أكثر من إجابة

ما هي علامات وأعراض الإصابة بالفيروس؟

- ☐ ارتفاع حرارة الجسم
- ☐ السعال
- ☐ الإسهال والقيء
- ☐ ضيق في التنفس
- ☐ العطس

ينتقل الفيروس من خلال:

- ☐ السعال و العطس
- ☐ لمس الأسطح المصابة
- ☐ عن طريق الدم
- ☐ التواجد في أماكن مزدحمة

ما هي طرق الحماية؟

- ☐ اغسل يديك بانتظام بالماء والصابون ، أو نظفهما بالكحول
- ☐ حافظ على مسافة متر واحد على الأقل بينك وبين الأشخاص المصابين بالسعال أو العطس
- ☐ تجنب لمس وجهك
- ☐ قم بتغطية فمك وأنفك عند السعال أو العطس
- ☐ ابق في المنزل إذا شعرت بتوعك
- ☐ الامتناع عن التدخين والأنشطة الأخرى التي تضعف الرئتين
- ☐ تجنب السفر غير الضروري
- ☐ الابتعاد عن مجموعات كبيرة من الناس

يمكن علاج عدوى فيروس كورونا باستخدام مضاد حيوي؟

- ☐ نعم
- ☐ لا

يوجد لقاح متوفر لـفيروس كورونا

- ☐ صح
- ☐ خطأ

من أين تحصل على معرفتك حول الفيروس.

☐ الموقع الرسمي لوزارة الصحة

☐ المجلات العلمية

☐ أطباء

☐ مواقع الجامعة

☐ برامج التلفاز و النشرات الاخبارية

☐ وسائل التواصل الاجتماعي (WhatsApp، Twitter، Instagram، Facebook)

إذا أجبت نعم على وسائل التواصل الاجتماعي, من أي منصة تحصل على المعلومات؟

☐ Facebook

☐ Instagram

☐ Twitter

☐ WhatsApp

☐ أخرى

هل تعتقد أن فيروس كورونا هو جزء من نظرية المؤامرة العالمية؟

☐ نعم

☐ لا

☐ ربما

هل تلتزم بقواعد الحجر الصحي الحكومي وتبقى في المنزل؟

☐ نعم

☐ لا

أجب عن الأسئلة المتعلقة بما تشعر به خلال الأسبوعين الماضيين من الحجر الصحي

	كل يوم تقريبا	أكثر من نصف الأيام	عدة أيام	لا على الاطلاق
هل تشعر بالتوتر أو القلق ؟	☐	☐	☐	☐
لديك مشكلة في الاسترخاء و الشعور بالراحة.	☐	☐	☐	☐
تقلق كثيرا بشأن أعداد المصابين بالفيروس.	☐	☐	☐	☐
عدم القدرة على الاستمرار بالأعمال اليومية.	☐	☐	☐	☐
عدم التوقف عن التفكير	☐	☐	☐	☐
تشعر بالإنزعاج بسرعة في فترة الحجر	☐	☐	☐	☐
الشعور بالخوف من تحول الوباء الى كارثة عالمية.	☐	☐	☐	☐

شكرا
